# In memory of Jerry Simpson 1939–2020

**DOI:** 10.1186/s40673-020-00113-1

**Published:** 2020-05-04

**Authors:** Chris I. de Zeeuw, Robert A. Hensbroek, Jun Maruta, Jan Voogd

**Affiliations:** 1 Department of Neuroscience, Erasmus MC, Dr. Molenwaterplein 40, 3015 GE Rotterdam, The Netherlands; 2 Department of Neuroscience and Physiology, NYU Langone Health, 550 First Avenue, New York, NY 10016 USA; 3 Department of Neurology, Icahn School of Medicine at Mount Sinai, 1 Gustave L. Levy Pl, New York, NY 10029 USA

It saddens us to announce the death of John “Jerry” I. Simpson (80), Professor Emeritus at the Department of Neuroscience and Physiology, NYU Langone Health, in New York. He passed away quietly on March 22nd after having been continually hospitalized for 3 months. Jerry is survived by his wife Diane.

Jerry was well known in the cerebellar community for his intellect, his encyclopedic knowledge, his outgoing personality and wit, his entertaining lectures sprinkled with humor, and his thought-provoking questions.

After getting his Ph.D. at MIT, he started in the cerebellar field as a postdoc with Masao Ito at the University of Tokyo. Subsequently, he was a postdoc with Rodolfo Llinas at the University of Iowa, and later joined the same department as a faculty member before the group permanently relocated to NYU. His research centered around single-cell neurophysiology of cerebellar neurons and their relation to the control of eye movements.

One of his first major contributions was the discovery that direction-specific retinal image slip modulates climbing fiber activity in the vestibulo-cerebellum [1, 2]. These visual climbing fiber signals are believed to contribute to error feedback processing during adaptation of the vestibulo-ocular reflex, and thereby form a crucial component of the Marr – Albus – Ito hypothesis on cerebellar learning [3–5].

He then went on to establish that the accessory optic and vestibular systems are complementary detectors of self-motion that share a reference coordinate framework defined by head movements relative to space [6]. Building on this knowledge, he showed that retinal slip signals close the vestibulo-ocular reflex feedback loop using common, mutually orthogonal axes in three-dimensional space [7–10]. The notion that movement is coded in the brain using a common coordinate frame was consistent with the observation he and colleagues had made about the geometries of extraocular muscles and semicircular canals closely aligning with each other across species, be it lateral- or frontal-eyed [11]. Jerry further discovered that this reference frame had an anatomical counterpart in the modular organization of the vestibulo-cerebellum [12–14] and that topographic arrangements were preserved in the projections to the vestibular and cerebellar nuclei from these cerebellar microzones [12, 15].

He also made important contributions to our current understanding on the information content of climbing fibers [16]. Indeed, he was one of the first to show that visual climbing fibers in the flocculus can also signal non-visual information [17, 18]. Moreover, he showed that low-frequency climbing fiber input transmits graded signals to Purkinje cells in terms of the number of high-frequency spikelets [19], calling for a re-examination of the widely-held notion that climbing fiber input results in an all-or-none response in a Purkinje cell.

The final stages of his career were primarily devoted to understanding the contribution of cortical interneurons in transforming mossy fiber activity to Purkinje cell output. He improved our insight into what unipolar brush cells, granule cells, Golgi cells, and molecular layer interneurons can encode during different forms of visual and vestibular stimulation [20–22].

Jerry was an extremely warm supervisor for many students and postdocs, taking care of minute details in person. Moreover, he was much interested in broadening opportunities for young investigators and donated generously to scientific organizations to fund scholarships and financial aid for meeting attendance.

Even though working had become physically taxing, Jerry remained an active scientist until a few months before his death and even managed to visit the last (2019) SfN meeting in Chicago, which he greatly enjoyed.

Jerry is buried in our blood and brain; he will be missed.

Chris De Zeeuw, Robert Hensbroek, Jun Maruta and Jan Voogd.
